# NIR-II Absorbing Conjugated Polymer Nanotheranostics for Thermal Initiated NO Enhanced Photothermal Therapy

**DOI:** 10.3390/bios13060642

**Published:** 2023-06-12

**Authors:** Kaiwen Chang, Xiaolin Sun, Qiaofang Qi, Mingying Fu, Bing Han, Yang Zhang, Wei Zhao, Tianjun Ni, Qiong Li, Zhijun Yang, Chunpo Ge

**Affiliations:** 1 Key Laboratory of Medical Molecular Probes, Department of Medical Chemistry, School of Basic Medical Sciences, Xinxiang Medical University, Xinxiang 453003, China; 171065@xxmu.edu.cn (K.C.); 50200106010@stu.xxmu.edu.cn (X.S.); 181040@xxmu.edu.cn (Q.Q.); fmy163wy@163.com (M.F.); 17839885739@163.com (W.Z.); tjni@xxmu.edu.cn (T.N.); 2 Shandong Provincial Key Laboratory of Detection Technology for Tumor Markers, College of Medicine, Linyi University, Linyi 276005, China

**Keywords:** conjugated polymer, nanotheranostics, photothermal therapy, NIR-II, nitric oxide

## Abstract

Photothermal therapy (PTT) has received constant attention as a promising cancer treatment. However, PTT-induced inflammation can limit its effectiveness. To address this shortcoming, we developed second near-infrared (NIR-II) light-activated nanotheranostics (CPNPBs), which include a thermosensitive nitric oxide (NO) donor (BNN6) to enhance PTT. Under a 1064 nm laser irradiation, the conjugated polymer in CPNPBs serves as a photothermal agent for photothermal conversion, and the generated heat triggers the decomposition of BNN6 to release NO. The combination of hyperthermia and NO generation under single NIR-II laser irradiation allows enhanced thermal ablation of tumors. Consequently, CPNPBs can be exploited as potential candidates for NO-enhanced PTT, holding great promise for their clinical translational development.

## 1. Introduction

Cancer is one of the primary causes of death in the world, posing serious threats to human health and well-being [1,2]. Photothermal therapy (PTT), as a promising therapeutic approach, has attracted much attention for its excellent remote spatiotemporal control precision and non-invasive therapeutic properties [1,2,3]. Remarkably, PTT utilizes light-absorbing agents to convert photoenergy into heat for thermal ablation of cells [4,5,6,7]. More importantly, near-infrared (NIR) light is more beneficial than visible light for reducing light attenuation in tissues, which allows therapeutic drugs with high NIR absorbance to achieve better light penetration depth and lower toxicity to biological systems [8,9]. To date, various types of near-infrared inorganic nanomaterials have been investigated and applied for PTT, for instance, rare earth ion-doped nanocrystals, novel metal nanostructures, and tungsten oxide nanowires [10,11]. However, the potential long-term safety concerns of inorganic nanomaterials have seriously limited their potential clinical applications [12].

Consequently, organic materials have attracted wide interest [13]. Particularly, conjugated polymer nanoparticles (CPNs) fabricated from semiconducting polymers are promising for biomedical applications owing to their outstanding optical merits [14,15,16,17,18,19,20,21,22]. Notably, the metal-free nature of CPNs allows them to bypass toxicity problems caused by metal ions [23]. Simultaneously, CPNs often possess optical advantages superior to inorganic semiconductor nanoparticles [24]. Furthermore, CPNs can efficiently convert photoenergy into mechanical acoustic waves and heat, making them superior candidates for photoacoustic imaging and photothermal therapy [25,26,27]. In addition, compared with carbon nanotubes and gold nanorods, they have stronger light absorption ability and higher photothermal conversion efficiency, resulting in faster heating [28,29]. Based on these encouraging performances, CPN-based theranostic agents have been used as photothermal nanomodulators for cancer treatment [30,31,32,33,34]. To date, CPNs have been demonstrated for NIR-II PTT [35,36,37,38,39,40]. Nevertheless, therapy-induced inflammation can dramatically impair the therapeutic efficacy of PTT [41,42]. Therefore, it is of great significance for tumor treatment to further overcome the defects of photothermal therapy.

Currently, gas therapy is considered a very promising tumor treatment strategy due to its high therapeutic efficacy and biosafety [43,44]. Within the gasotransmitter family, NO plays extremely important physiological or pathophysiological roles in nearly every organ system [45,46,47]. The biological effect of NO is a double-edged sword [48]. At low physiological concentrations, NO can facilitate vasodilation and angiogenesis, thereby promoting tumor progression to some degree [49]. Conversely, at elevated concentrations, NO may exhibit inhibitory or toxic effects on cancer cells [50]. Thus, researchers have developed various NO donors [51,52,53]. Among these, N,N′-disecbutyl-N,N′-dinitroso-p-phenylenediamine (BNN6) was regarded as a powerful NO generator for gas therapy [54,55]. However, BNN6 has the shortcoming of UV-vis photoresponsivity, which seriously restricts its application and development. To overcome this deficiency, BNN6 exhibits near-infrared response through π-π superpositioning with other molecules [54]. More importantly, BNN6 is also a heat-responsive NO donor [56,57,58,59]. Therefore, it is of great necessity and importance to design and prepare a NO generator using conjugated polymer nanoparticles for photothermal/gas dual-modal precise therapy under NIR-II irradiation, to induce a synergistic effect by significantly inhibiting the growth of tumors.

Inspired by these studies, we report a smart nanotheranostics (CPNPB)-based conjugated polymer with a NIR-II light-activated NO generator for enhanced PTT. CPNPBs consist of three components: conjugated polymer (IN-NDI), NO donor (BNN6), and amphiphilic polymer (F127), and exhibit excellent biocompatibility. Meanwhile, CPNPBs possess broad absorbance in the NIR-II window and a photothermal conversion efficiency of 55.6%, enabling the generation of favorable heat under 1064 nm laser irradiation. In particular, the generated heat can also trigger the decomposition of BNN6 to produce NO. Benefiting from the efficient photothermal conversion and NO generation under single NIR-II laser irradiation, CPNPBs can achieve a significantly enhanced photothermal treatment effect. More importantly, this strategy can be applied to develop various thermally controlled therapeutic agents for enhanced cancer therapy.

## 2. Materials and Methods

### 2.1. Materials

4,9-Bis(5-bromo-2-thienyl)-2,7-bis(2-octyldodecyl)-benzo[lmn] [3,8] phenanthroline-1,3,6,8(2H,7H)-tetrone (NDI) was obtained from Derthon Optoelectronic Materials Science Technology Co., Ltd. (Shenzhen, China); 4,4′-Bis(octyloxy)-2,2′-bis(trimethylstannyl)-5,5′-bithiazole (IN) was procured from SunaTech Inc. (Suzhou, China). 3-[4,5-Dimethylthiazol-2-yl]-2,5-diphenyltetrazolium bromide (MTT), anhydrous tetrahydrofuran (THF, 99.9%), phosphate-buffered saline (PBS), acridine orange (AO), ethidium bromide (EB), and Pluronic F-127 were sourced from Sigma-Aldrich (Shanghai, China). Griess reagent kits were acquired from Beijing Solarbio Science & Technology Co., Ltd. (Beijing, China). Tetrakis(triphenylphosphine)palladium(0) (Pd[PPh_3_]_4_, 99%), (4,4,5,5-tetramethyl-1,3,2-dioxaborolan-2-yl)benzene, and chloroform-d were procured from J&K Chemical Ltd. (Beijing, China). Ultrapure water (18.25 MΩ/cm^2^ at 25 °C) was employed throughout the study. All other chemical reagents were utilized without further purification.

### 2.2. Characterization

The ^1^H NMR spectra were obtained with a Fourier-transform-mode 400 MHz Bruker NMR spectrometer using deuterated chloroform (CDCl_3_) as the solvent. A Waters gel permeation chromatography (GPC) 2410 system calibrated with polystyrene standards and trichloromethane (CHCl_3_) was used to determine the molecular weights and molecular weight distributions. A standard transmission electron microscope (TEM) (Hitachi H-600, Hitachi Ltd., Tokyo, Japan) was used to capture the images of the samples. Dynamic light scattering (DLS) and ζ-potential measurements were performed using the NanoBrook 90Plus Zeta (Brookhaven, NY, USA). A UV−vis 2600 spectrophotometer equipped with an ISR-2600Plus integrating sphere was employed to measure the absorption spectra over the wavelength range of 200 to 1200 nm.

### 2.3. Synthesis of Conjugated Polymer IN-NDI

The synthesis of IN-NDI polymer with a donor–acceptor (D-A) structure was carried out through Stille coupling polymerization. In a 25 mL single-necked flask, NDI (287.33 mg, 0.25 mmol) and IN (187.57 mg, 0.25 mmol) were dissolved in 10 mL of toluene and degassed with nitrogen (N2) using five freeze–pump–thaw cycles to eliminate air. Subsequently, Pd(PPh3)4 (5 mg, 0.004 mmol) was added as the catalyst, and the reaction mixture was subjected to heating at 110 °C for 48 h under a nitrogen atmosphere. To remove bromine end groups and stannyl end groups, 1 mL of (4,4,5,5-tetramethyl-1,3,2-dioxaborolan-2-yl)benzene (20 mg, dissolved in toluene) and 0.2 mL of bromobenzene were gradually added to the reaction mixture, respectively, and reaction was continued for 4 h,. The reaction mixture was subsequently cooled to ambient temperature, and added dropwise to methanol (150 mL) to obtain its precipitation, which was then filtered and washed with ammonia solution, deionized water, ethanol, and acetone. The precipitate was then redispersed in acetone (150 mL) and stirred for 24 h. The resulting product was collected by filtration and dried at 50 °C under vacuum to yield a brown-black solid (237.87 mg; 76%).

### 2.4. Synthesis of BNN6 for Nitric Oxide Donor

According to the previously reported method, the target compound N,N′-di-sec-butyl-N,N′-dinitroso-1,4-phenylenediamine (BNN6) was synthesized via an addition reaction [55]. Initially, N,N′-bis-sec-butylamino-p-phenylenediamine (BPA) (2.34 mL, 10 mmol) was dissolved in ethanol (18 mL). Subsequently, a deoxygenated NaNO_2_ solution (20 mL, 6 M) was added under an inert nitrogen atmosphere with constant stirring. After 30 min, HCl (20 mL, 6 M) was added dropwise into the reaction mixture, which changed from red to orange in color and formed a beige precipitate. Stirring for another 4 h, the product was collected through centrifugation, washed with water and 50% *v*/*v* ethanol/water to remove residual reagents, and vacuum dried overnight in the dark. The structure of BNN6 was validated by ^1^H NMR spectroscopy and mass spectrometry.

### 2.5. Preparation of CPNPs and CPNPBs

To prepare the nanoparticles, the reprecipitation method was utilized [60]. Initially, solutions of polymer, BNN6, and F127 were prepared in tetrahydrofuran with concentrations of 1 mg/mL, 1 mg/mL, and 10 mg/mL, respectively. These solutions were then combined at a volume ratio of 1:1:2 and stirred vigorously. Subsequently, 2 mL of the mixture solution was rapidly injected into 10 mL of deionized water and sonicated for 3 min in an ice–water bath. The tetrahydrofuran was removed by rotary steaming, and the remaining nanoparticles were purified using a 0.22 μm filter membrane to eliminate larger particles. The nanoparticles were concentrated as necessary and stored in a refrigerator.

### 2.6. Photothermal Performance of CPNPs and CPNPBs

The photothermal effects of CPNPs and CPNPBs were examined by monitoring the temperature changes under 1064 nm laser irradiation for 600 s. The dependence on optical power density was investigated by exposing the solution to a near-infrared laser with varying optical power densities. Additionally, the concentration dependence was studied by using solutions with different concentrations but the same optical power densities. To determine the photothermal conversion efficiency, 0.5 mL of nanoparticles were added to an EP tube and exposed to a 1064 nm laser with a power density of 0.6 W/cm^2^ until equilibrium was achieved. After turning off the laser, the solution was cooled to ambient temperature, and the maximum temperature (T_max_) and the initial temperature (T_min_) were recorded. The photothermal conversion efficiency (η) was determined using the following equation:

η = [hs(T_max_ − T_min_) − Q_dis_]/I(1 − 10^−A1064^) (1)

where η is the heat transfer coefficient, s is the surface area of the container, Q_dis_ is the heat lost by water, I is the incident laser power (0.6 W/cm^2^), and A_1064_ is the absorbance of CPNPs (CPNPBs) at 1064 nm. The value of hs was determined using the equation:

hs = cm/τ_s_ (2)

in which τ_s_ is the heat transfer time constant of the solution, m is the mass of deionized water, and c is the heat capacity of water. The photothermal conversion efficiencies of CPNPs and CPNPBs were found to be 46% and 55.6%, respectively.

### 2.7. NO Detection In Vitro

#### 2.7.1. Standard Curve of Nitric Oxide

The Griess reagent is capable of oxidizing nitric oxide into nitrite, subsequently forming a pink diazo compound that exhibits increased UV-visible absorbance at 540 nm. First, 1 M NaNO_2_ standard solution was diluted to various concentrations (5–60 μM). Then, 900 μL of the mixture of Griess reagent I and II (Griess reagent I:Griess reagent II = 900:1) and 900 μL different concentrations of NaNO_2_ solution were uniformly mixed, respectively. Subsequently, the response of the Griess reagent to NO was evaluated. The standard curve of the absorption value at 540 nm with different concentrations of NaNO_2_ was obtained.

#### 2.7.2. NO Detection of CPNPBs In Vitro

The NO-releasing property of nanoparticles was measured using the Griess reagent kit. The CPNPB aqueous solution (100 μg/mL) was placed in a 2 mL centrifuge tube and subjected to irradiation by a 1064 nm laser, which resulted in the release of NO into the supernatant. At each fixed time, 150 μL of nanoparticles were added to a 96-well plate, followed by adding 150 μL of the mixture of Griess reagent I and II (Griess reagent I:Griess reagent II = 900:1). Absorbance at a specific wavelength of 540 nm was measured utilizing a microplate reader. The released NO amount was calculated based on the NO concentration standard curve obtained.

### 2.8. In Vitro Cytotoxicity by MTT Assay

The cytotoxicity of nanoparticles in vitro was measured using an MTT assay. MCF-7 cells and HeLa cells were obtained from Zhongqiao Xinzhou Company (Shanghai, China) and grown in DMEM cell medium containing 10% FBS, 1% penicillin, and 1% streptomycin. The cells were incubated overnight at 37 °C in a 5% carbon dioxide incubator. The original medium was then removed and replaced with DMEM containing various concentrations of nanoparticles (12.5 to 200 μg/mL). After incubation for another 12 h, the cells were irradiated with or without a 1064 nm laser (600 mW/cm^2^) for 10 min. After 24 h of incubation at 37 °C in the dark, 20 μL MTT stock solution (5 mg/mL in sterile PBS) was added. After 4 h, the medium was replaced with 100 μL dimethyl sulfoxide. Cell survival was calculated by measuring absorption at 490 nm utilizing a microplate reader (BioTeK PowerWave XS, BioTek Instruments, Inc., Winooski, VT, USA).

AO/EB tests: HeLa cells were cultured for 12 h in 12-well plates with the same cell density. Subsequently, the cells were then subjected to various treatments, including PBS; PBS + laser; CPNPs; CPNPs + laser; CPNPBs; and CPNPBs + laser (laser: 1064 nm, 0.6 W/cm^2^, 10 min). Subsequently, the treated cells were incubated at 37 °C for 24 h, followed by three washes with PBS. A pre-prepared fluorescent dye, AO/EB, was added to each well and shaken gently to ensure even distribution. Finally, the stained cells were examined and imaged utilizing a fluorescence microscope.

### 2.9. In Vivo Antitumor Activity and Biosafety

For the in vivo experiments, female Balb/c mice aged 5 to 6 weeks were obtained from Huaxing Experimental Animal Farm (Zhengzhou, China). The animal studies were approved by the Animal Management and Ethics Committee of Xinxiang Medical University. Two weeks prior to the commencement of treatment, 5 × 10^6^ 4T1 cells were inoculated into the right hindlimbs of each mouse. Once the size of tumors reached 90–120 mm^3^, these mice were segregated into six groups (n = 5). Each group received a different treatment: (1) PBS; (2) PBS + laser; (3) CPNPs; (4) CPNPs + laser; (5) CPNPBs; and (6) CPNPBs + laser. The laser irradiation was set at 1064 nm and 0.6 W/cm^2^ for 10 min. The nanoparticle concentration was maintained at 100 μg/mL and the injection dose administered was 1.5 mg/kg per mouse. During treatment, tumor volumes and mouse body weights were monitored every other day. After a period of 28 days, the mice were euthanized for further assessment. Tumor tissues and vital organs were extracted for H&E staining analysis. Microscopic images of the tissue sections were captured. Tumor dimensions were recorded every other day and volume was computed using the formula: V = L/2 × W^2^.

## 3. Results and Discussion

### 3.1. Synthesis and Characterization of Nanoparticles

**Figure 1:**
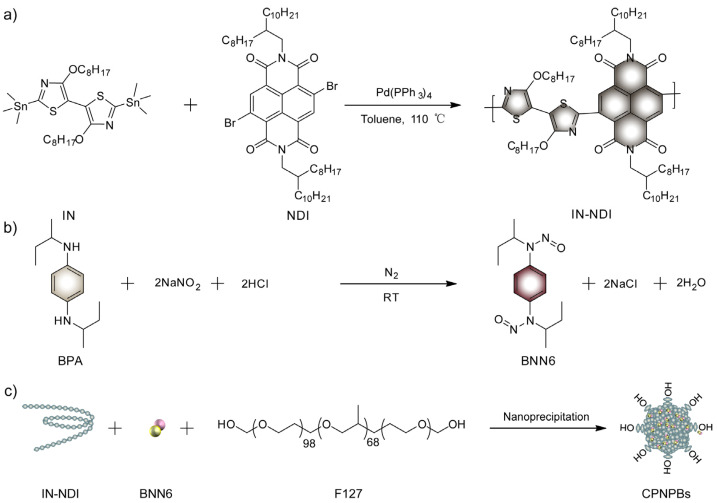
(**a**) Synthetic route of conjugated polymer IN-NDI. (**b**) Synthetic route of BNN6. (**c**) Preparation of CPNPBs.

In this study, a novel conjugated polymer IN-NDI was synthesized via a Stille coupling reaction, as depicted in Figure 1a, and the chemical structures of IN-NDI were verified using ^1^H NMR spectroscopy (Figure S1, Supplementary Materials). GPC analysis of the IN-NDI revealed an Mn of 3.5 kDa and a polydispersity index (Mw/Mn) of 2.8 (Figure S2). Moreover, the synthesis of BNN6 was accomplished through a previously established method (Figure 1b). The structure of purified BNN6 was confirmed to be correct by ^1^H NMR and FT-IR (Figures S3 and S4). The nanoparticles were prepared through the nano reprecipitation method [60], utilizing F127 as a functional amphiphilic polymer (Figure 1c). The distinction between the two types of nanoparticles hinges upon their composition, with BNN6 being present in CPNPBs while absent from CPNPs.

**Figure 2:**
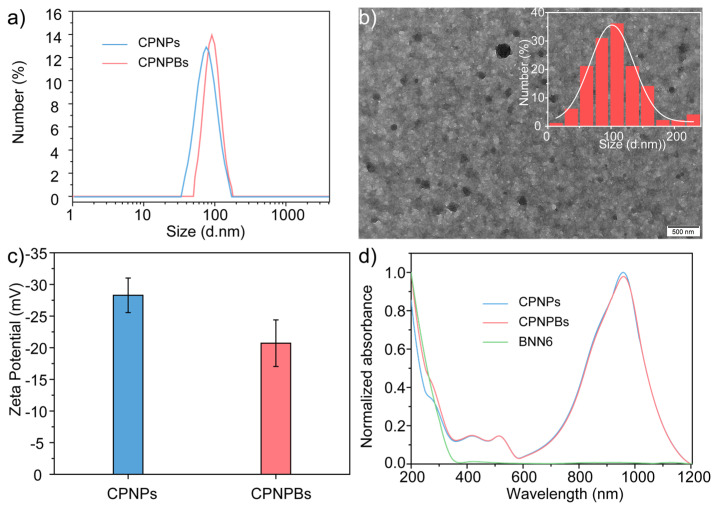
Characterization of CPNPBs. (**a**) Representative DLS profiles of CPNPs and CPNPBs in water. (**b**) Representative TEM images of CPNPBs. (**c**) The ζ-potential of the CPNPs and CPNPBs. (**d**) UV-vis-NIR spectra of BNN6, CPNPs and CPNPBs.

Dynamic light scattering (DLS) analyses revealed that the average hydrodynamic diameters of CPNPs and CPNPBs were approximately 80 nm and 120 nm, respectively (Figure 2a). Transmission electron microscopy (TEM) confirmed the spherical morphology of both CPNPBs and CPNPs and showed average diameters of 70 nm and 110 nm, respectively (Figure 2b and Figure S7). Additionally, CPNPBs and CPNPs exhibited large zeta potentials of −21 mV and −28 mV (Figure 2c), respectively, due to the presence of F127 (Figure S8a), resulting in high nanoparticle stability under physiologically relevant conditions (Figure S8b,c). The absorption spectra of CPNPs, CPNPBs, and BNN6 are shown in Figure 2d, in which CPNPs and CPNPBs have broad absorption in the NIR-II window, making them suitable as photothermal agents for NIR-II photothermal therapy.

### 3.2. Photothermal Properties of CPNPs and CPNPBs

**Figure 3:**
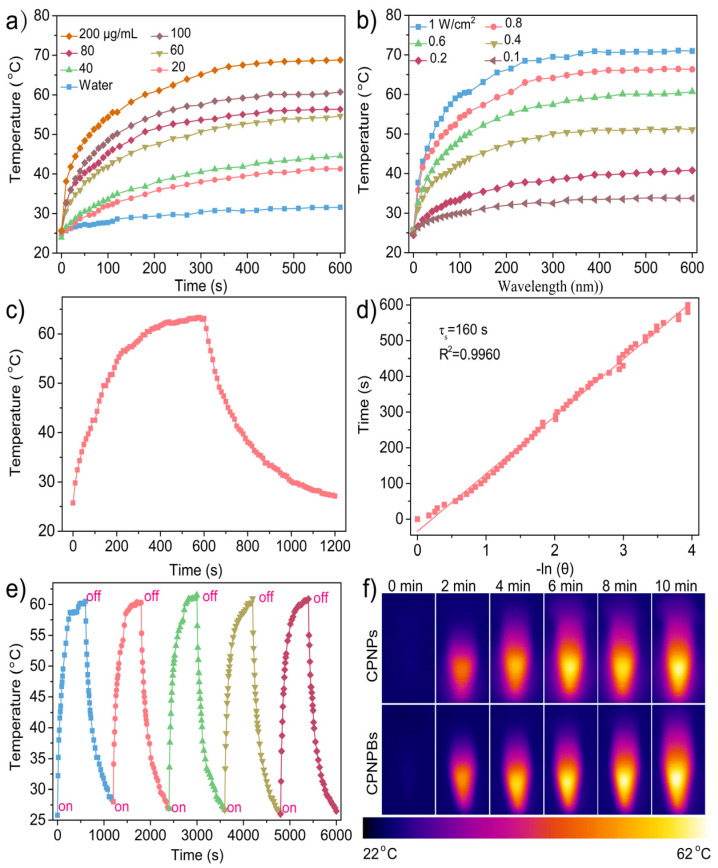
Photothermal properties of CPNPBs. (**a**) Concentration-dependent photothermal curves of CPNPBs under 1064 nm laser irradiation. (**b**) Photothermal heating curves of CPNPB dispersions (100 μg/mL) irradiated using a 1064 nm laser at varied power densities (0.1, 0.2, 0.6, 0.8, 1.0 W/cm^2^). (**c**) Photothermal effect of CPNPB aqueous solution (100 μg/mL) excited with 1064 nm laser. (**d**) Linear relationship curves between time (s) versus −ln θ based on panel e. (**e**) Temperature elevation of CPNPB dispersion under five on/off cycles. Each color represents a on/off cycle. (**f**) Photothermal IR images of CPNPs and CPNPBs under 1064 nm laser irradiation for 10 min.

For full evaluation of the photothermal conversion performance of CPNPBs and CPNPs, the temperature curves of CPNPBs and CPNPs solutions at varying concentrations (0–100 μg/mL) under 1064 nm laser irradiation at 0.6 W/cm^2^ are depicted in Figure 3a and Figure S9a. The results show that the highest temperature reached 59.8 °C after 10 min irradiation and the maximum temperature of the solution decreased with the decrease of the concentration of the solution. Furthermore, the temperature of CPNPs and CPNPBs solutions varied with the different laser power densities (0.2, 0.4, 0.6, 0.8, and 1 W/cm^2^) (Figure 3b and Figure S9b). We found that the effects of CPNPs and CPNPBs showed strong dependence on the density of the near-infrared laser power. In addition, the photothermal conversion efficiency is one of the important parameters for assessing photothermal conversion capability. We further measured the temperature change in the nanoparticle solution under a laser (1064 nm, 0.6 W/cm^2^). The laser was shut off when the temperature approached a stable level after irradiation for 600 s (Figure 3c and Figure S9c). At a concentration of 100 μg/mL nanoparticles, the solution temperature rapidly increased to the mixture temperature after laser irradiation for 600 s. It can be seen from Figure 3d and Figure S9d that the photothermal conversion efficiencies of CPNPs and CPNPBs are 46% and 55.6%, respectively. To evaluate the photothermal stability of CPNPs and CPNPBs solutions, the recyclable temperature changes of CPNPs and CPNPBs are shown in Figure 3e and Figure S9e. CPNPs and CPNPBs had stable photothermal conversion capabilities throughout five consecutive heating/cooling cycles. Then, we used the infrared thermal image to visualize the temperature changes of different solutions under 0.6 W/cm^2^ laser irradiation. We can observe that the temperature changes of BNN6 and PBS were not obvious (Figure S9f). Figure 3f shows that the maximum temperature of CPNPBs and CPNPs reached 64 °C and 59 °C, respectively, which meets the heat dissipation requirements of PTT. These results suggest that CPNPBs can be a viable candidate for photothermal therapy.

### 3.3. NO Release Performance of CPNPBs

**Figure 4:**
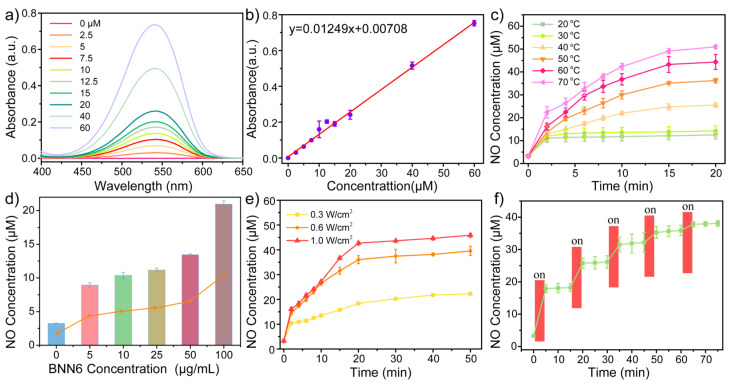
(**a**) The absorption spectra of the Griess agent to various concentrations of NaNO_2_. (**b**) The response curve of Griess agent to NO. (**c**) NO release of CPNPBs (100 μg/mL) in a water bath at a series of temperatures (20, 30, 40, 50, 60, 70 °C) for 20 min. (**d**) NO release of CPNPBs loaded with different concentrations of BNN6 under 1064 nm laser irradiation (0.6 W/cm^2^) for 10 min. (**e**) NO release curves of CPNPBs (100 μg/mL) under varying power densities of irradiation using a 1064 nm laser. (**f**) NO controlled release curve of CPNPBs under a switching 1064 nm laser irradiation (0.6 W/cm^2^).

As mentioned above, CPNPBs are excellent photothermal agents with potential application in photothermal therapy. Under 1064 nm near-infrared laser irradiation, the heat generated from the polymer causes BNN6 to release NO. We used the classical Griess method to quantitatively study the release behavior of NO in CPNPBs [5], and verified that the production of NO is regulated by light. The response of the Griess reagent to NO was evaluated and the results are depicted in Figure 4a. A standard curve for calculating NO concentrations was established using NaNO_2_ standard samples ranging from 0 μM to 60 μM (Figure 4b). To verify that NO release from CPNPBs is a response to temperature, the amount of NO released from CPNPBs was evaluated at multiple temperatures ranging from 20 °C to 70 °C over 20 min (Figure 4c). At temperatures below 30 °C, BNN6 underwent slight decomposition and released a small quantity of NO. As the temperature rose to 50 °C, the rate of NO release increased. Therefore, relatively modest thermal energy (up to 50 °C) directly induces the structural decomposition of BNN6 to yield nitric oxide. Afterwards, we adjusted the concentration of IN-NDI in CPNPBs to 100 μg /mL, and loaded different amounts of BNN6 (5, 10, 25, 50, and 100 μg /mL) in CPNPB solution. Upon exposure to 1064 nm excitation light (0.6 W/cm^2^) for 10 min, the amount of NO released increased with increasing BNN6 concentration (Figure 4d). With a BNN6 loading level of 100 μg/mL, the NO release amount could reach 20.9 μg /mL. Furthermore, the relationship between the release of NO from CPNPBs and the optical power density of near-infrared excitation was also examined (Figure 4e). The amount of NO released increased with increasing optical power, further demonstrating that the NO release of the CPNPB solution is dependent on power density.

The controllability of NO release using near-infrared light was examined. Upon exposure to near-infrared light, NO was rapidly released. When the light was turned off, NO release slowed significantly due to slightly delayed release (Figure 4f). This indicates that the CPNPBs solution exhibits excellent near-infrared light controllability for NO release. Through modulation of near-infrared light switching and intensity, the NO concentration can be adeptly regulated as needed, which is imperative for retaining NO levels within the therapeutic index and reducing the risk of NO toxicity.

### 3.4. In Vitro Cytotoxicity Testing

**Figure 5:**
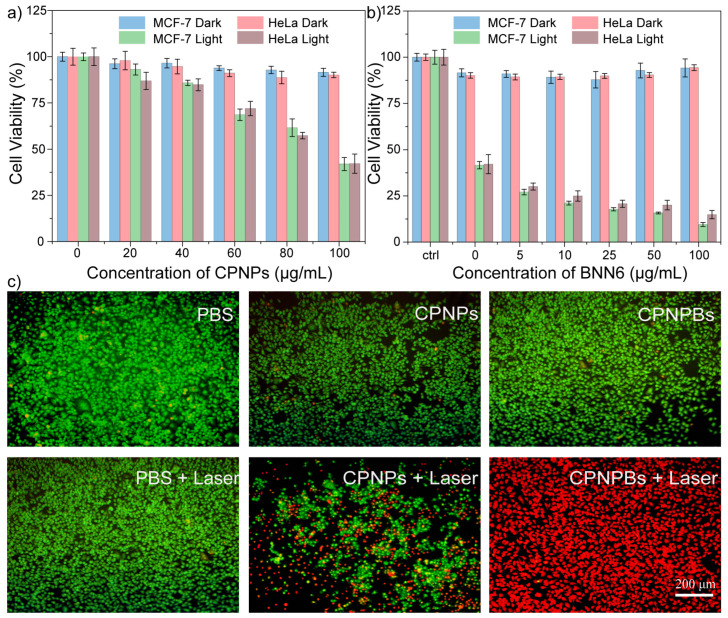
The cellular test of CPNPs and CPNPBs. (**a**) MTT assay of HeLa cells and MCF-7 cells treated with CPNPs at various concentrations of 0, 20, 40, 60, 80, and 100 μg/mL with or without light for 5 min to assess the viability of the cells. Error bars denote the standard deviation (n = 6). (**b**) MCF-7 cells and HeLa cells were treated with different concentrations of BNN6 (0~100 μg/mL) with or without light for 5 min to assess the cells’ viability. Error bars denote the standard deviation (n = 6). (**c**) Fluorescent images of live/dead cell staining of HeLa cells under different treatment conditions. Green and red represent live and dead cells, respectively. Scale bar = 200 μm.

CPNPBs have demonstrated potential as NO donors, but their cytotoxicity requires further investigation. MCF-7 and HeLa cells were incubated with varying concentrations of CPNPs and CPNPBs, respectively. Cellular viability was evaluated utilizing the MTT assay. As depicted in Figure 5a, cell viability exceeded 85% in the absence of light, indicating that CPNPs exhibit negligible cytotoxicity under dark conditions. However, upon exposure to 1064 nm excitation light (0.6 W/cm^2^) for 5 min per well, cell viability exhibited a significant downward trend. The survival rate of cells incubated with 100 μg/mL CPNPs was reduced to 41%. Furthermore, CPNPBs solution can produce NO that kills cancer cells under light conditions (Figure 5b). With increased BNN6 loading (5–100 μg/mL) in CPNPBs, cell survival decreased from 40% to 9% under laser exposure. Therefore, it can be concluded that the dual effect of the photothermal activity and NO will produce a better therapeutic effect.

To further detect the photothermal toxicity of CPNPBs, the apoptosis of HeLa cells was detected using the AO/EB (acridine orange/ ethidium bromide) double fluorescent dye method. The results are shown in Figure 5c; CPNPs and CPNPBs exhibited no obvious cytotoxicity in the absence of light. Under light conditions, cytotoxicity was significantly enhanced after treatment with CPNPBs compared with the control groups. These outcomes are consistent with the aforementioned MTT assay results, indicating that photothermal and NO synergistic therapy could produce a better therapeutic effect than single thermal therapy.

### 3.5. In Vivo Tumor Eradication and Biosafety of CPNPBs

**Figure 6:**
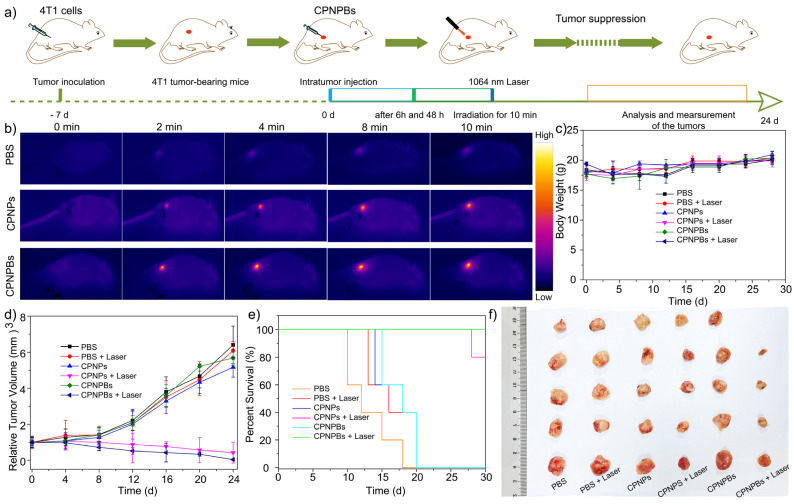
The pharmacodynamic evaluation of CPNPBs in vivo. (**a**) Time schedule of CPNPB-mediated synergistic cancer therapy and side-effect study. (**b**) IR thermal images of orthotopic 4T1 tumor-bearing mice during photoirradiation (1064 nm) at 24 h post-injection of CPNPBs, CPNPs, or PBS for 10 min. Laser conditions: 0.6 W/cm^2^. (**c**) The body weight changes of mice after various treatments over 28 days. Each data point represents the mean ± standard deviation from n = 5 animals. (**d**) The relative tumor volumes curves. Each data point denotes the mean ± standard deviation from n = 5 animals. (**e**) Survival curves of various groups of tumor-bearing mice after different treatments (n = 5). (**f**) Photograph of the dissected tumors after different treatments.

In vivo assessment of the combinatorial antitumor efficacy of CPNPBs was performed in BALB/c mice bearing 4T1 mammary tumors. Thirty BALB/c mice with 4T1 mammary tumors were separated into six groups and treated with (1) PBS, (2) PBS, (3) CPNPs, (4) CPNPs, (5) CPNPBs, and (6) CPNPBs, respectively. Groups one, three, and five were not subjected to 1064 nm laser irradiation. In contrast, the remaining groups were exposed to 1064 nm laser irradiation (0.6 W/cm^2^) for 10 min at 6 h and 48 h after administration. Tumor volumes and body weights of all mice were measured at regular intervals over 28 days, and tumor samples were obtained on the 28th day. Figure 6a shows the schematic illustration of the anticancer treatment process. The thermal response of the mice to the treatments was evaluated using infrared imaging. As depicted in Figure 6b, intratumoral injection of PBS resulted in an increase of tumor temperature to only 38.8 °C, whereas injection of CPNPs or CPNPBs led to rapid elevation of temperature, which reached a maximum of approximately 65 °C upon laser irradiation. These findings indicate that CPNPs and CPNPBs have good photothermal effects in vivo. As indicated in Figure 6c, all nanomaterials exhibited adequate safety profiles based on the absence of significant weight loss under either dark conditions or 1064 nm laser irradiation. The tumor volume of mice was monitored for 28 days (Figure 6d). Compared with other treatment groups, the tumor volume of the CPNPBs group and CPNPs group irradiated by NIR-II laser were significantly reduced. Meanwhile, the reduction in tumor volume was much more significant in the CPNPBs group than in the CPNPs group, which indicates that the effect of photothermal/gas therapy was superior to that of photothermal therapy alone.

More significantly, the average lifespan of mice in the four control groups was determined to be 16–20 days (Figure 6e), much shorter than that of mice in the CPNPs + laser or CPNPBs + laser groups. Furthermore, mice in the CPNPBs + laser group exhibited a longer average lifespan than those in the CPNPs + laser group, further demonstrating the superior antitumor efficacy of CPNPBs. Figure 6f shows representative photographs of the dissected tumors after different treatments. Tumor sizes in the CPNPBs + laser group were significantly smaller than those in other groups, further confirming the outstanding synergistic therapy effect of CPNPBs with laser irradiation. In a word, mice treated with CPNPBs and laser irradiation exhibited the greatest tumor inhibition compared with control groups.

**Figure 7:**
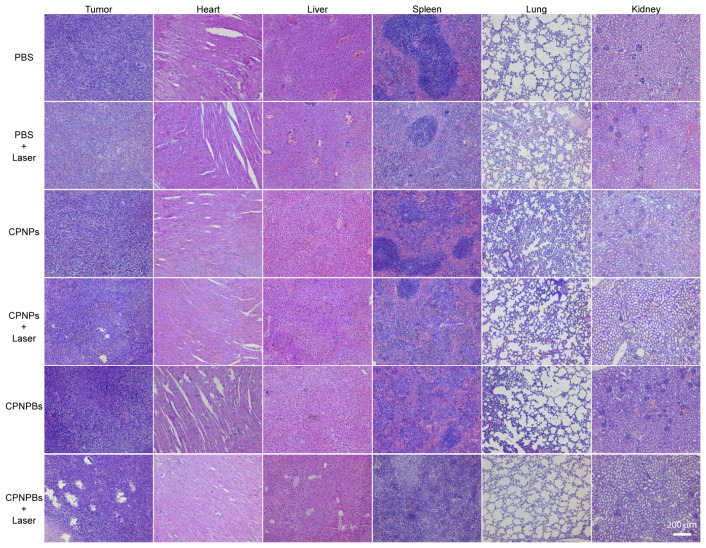
H&E staining images of major organs from different groups after different treatments. Scale bar: 200 µm.

To further confirm the therapeutic effectiveness of CPNPBs in vivo, H&E staining was used to assess the histological damage to tumor sections and major metabolic organs in mice following various treatments (Figure 7). Results showed that the CPNPBs + laser group exhibited significantly higher levels of tumor cell fragmentation compared with other groups. Additionally, no significant physiological or morphological damage was observed in any treatment group. These findings suggest that CPNPBs demonstrate exceptional theranostic potential for PTT/NO against tumors while maintaining excellent biological safety.

## 4. Conclusions

In this study, we constructed a type of nanotheranostics (CPNPBs) based on a conjugated polymer to synergistically combine PTT and gas therapy against cancer. CPNPBs not only possess excellent photothermal conversion performance because of broad absorbance in the NIR-II window for PPT, but also controllably release NO generated by NIR-II light activation for gas therapy. In addition, the CPNPBs show excellent biocompatibility and high photothermal conversion efficacy up to 55.6%. Furthermore, the generated heat can also initiate the decomposition of BNN6 to produce NO to enhance significantly the photothermal treatment effect of CPNPBs. The proposed approach offers a promising strategy to address the limitations of traditional photothermal therapy. Moreover, by incorporating other therapeutic molecules into these nanocomposites, this strategy can be applied to develop various intelligent controlled therapeutic agents for enhanced cancer therapy.

## Data Availability

Not applicable.

## Supplementary Materials

The following supporting information can be downloaded at: https://www.mdpi.com/article/10.3390/bios13060642/s1, Figure S1: 1H NMR spectra of IN-NDI polymer; Figure S2: Gel permeation chromatography (GPC) of IN-NDI polymer; Figure S3: 1H NMR spectra of the product BNN6; Figure S4: The MS of synthesized BNN6; Figure S5: FT-IR spectra of BNN6; Figure S6: The photodecomposition route of BNN6 with 365 nm UV irradiation and the color change during decomposition (digital pictures); Figure S7: Representative TEM images of CPNPs; Figure S8: Hydrodynamic diameters of nanoparticles as a function of storage at room temperature; Figure S9: Photothermal properties of CPNPs.
