# Supplementary material for: NIR-II Absorbing Conjugated Polymer Nanotheranostics for Thermal Initiated NO Enhanced Photothermal Therapy
Source: Biosensors (Basel). 2023 Jun 12;13(6):642. doi: 10.3390/bios13060642 (PMC10296163; doi:10.3390/bios13060642)
Supplement: Supplementary file 1 [file biosensors-13-00642-s001.zip › biosensors-2418000-supplementary.pdf]

biosensors

MDPI

Supporting Information

NIR-II Absorbing Conjugated Polymer Nanotheranostics for Thermal Initiated NO Enhanced Photothermal Therapy

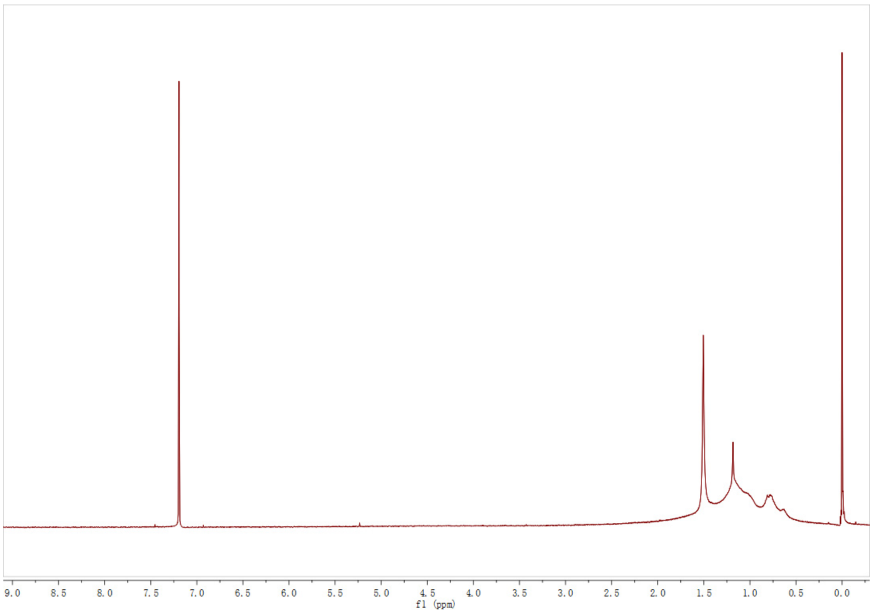

Figure S1. ^{1}H NMR spectra of IN-NDI polymer.

GPC Results

	Distribution Name	Mn (Daltons)	Mw (Daltons)	MP (Daltons)	Mz (Daltons)	Mz+1 (Daltons)	Polydispersity	Mz/Mw	Mz+1/Mw
1		35088	99343	33355	281707	529074	2.831265	2.835693	5.325719

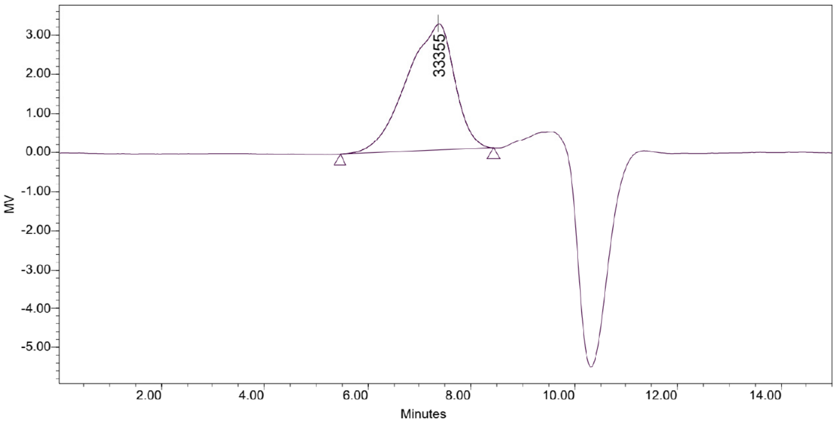

Figure S2. Gel permeation chromatography (GPC) of IN-NDI polymer.

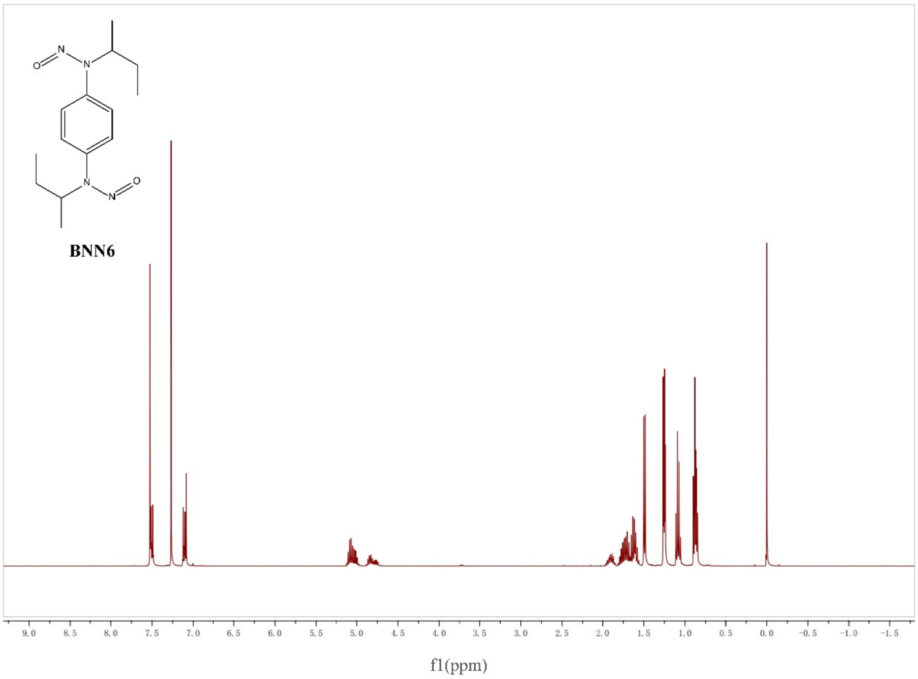

Figure S3. ^{1}H NMR spectra of the product BNN6: δ = 7.52 (4H), 4.95-4.69 (2H), 2.00-1.84 (2H), 1.81-1.69 (2H), 1.48 (t, J=7.6Hz, 6H), 1.08(td, J=7.4, 5.3Hz, 6H).

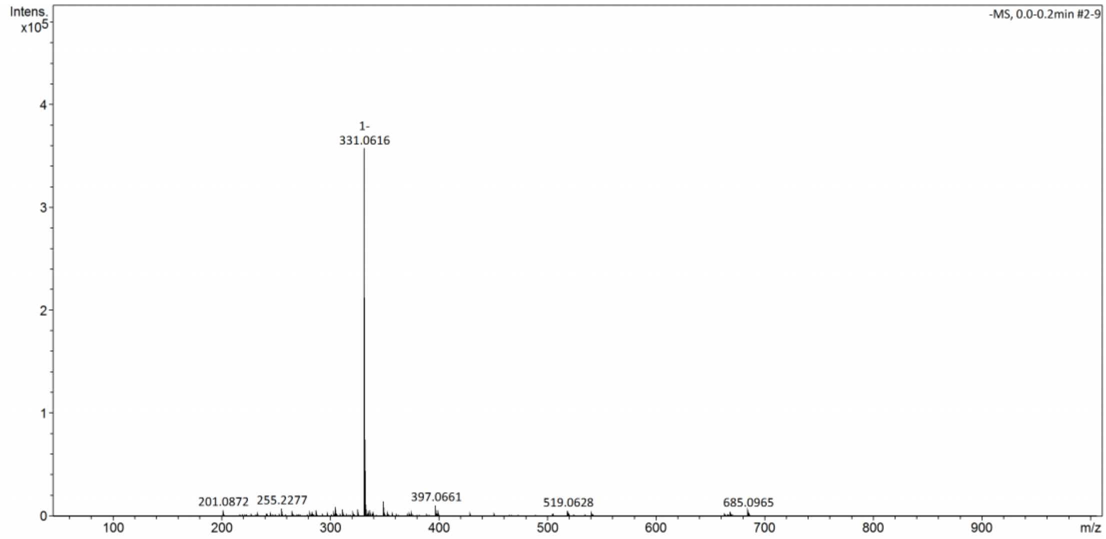

Figure S4. The MS of synthesized BNN6.MS (ESI^{+}): calcd for $C_{14}H_{22}N_{4}O_{2}Na$, 301.1635 $[M + Na]^{+}$; found 301.1631 $[M + Na]^{+}$.

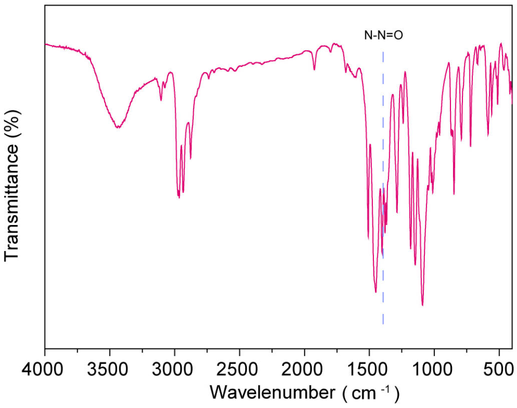

Figure S5. FT-IR spectra of BNN6: the feature N-N=O vibration band at the wavenumber of 1451 cm^{-1}.

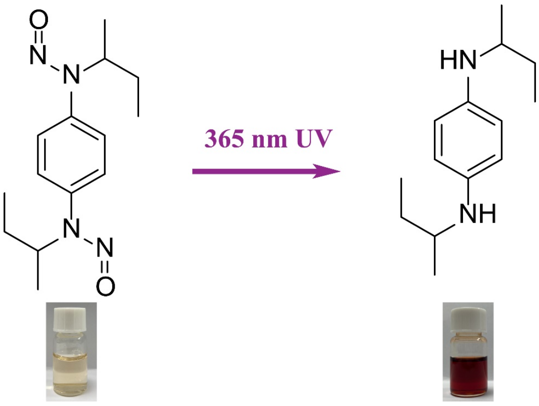

Figure S6. The photodecomposition route of BNN6 with 365 nm UV irradiation and the color change during decomposition (digital pictures).

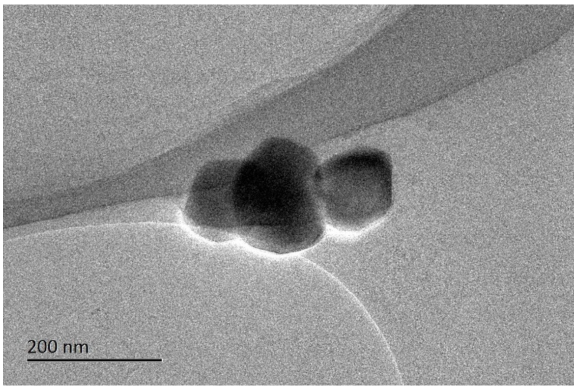

Figure S7. Representative TEM images of CPNPs.

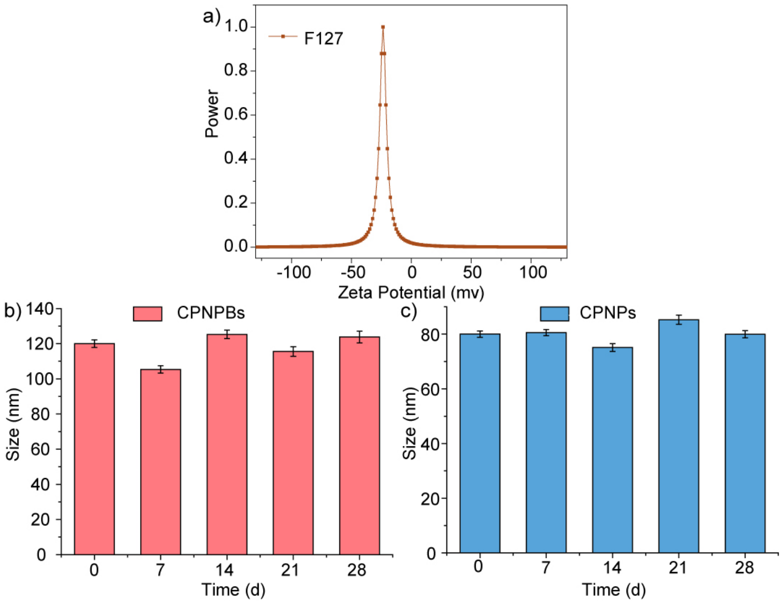

Figure S8. (a)The ζ-potential of pure F127. (b)The hydrodynamic diameter of CPNPBs versus the storage time at room temperature. (c) The hydrodynamic diameter of CPNPs versus the storage time at room temperature.

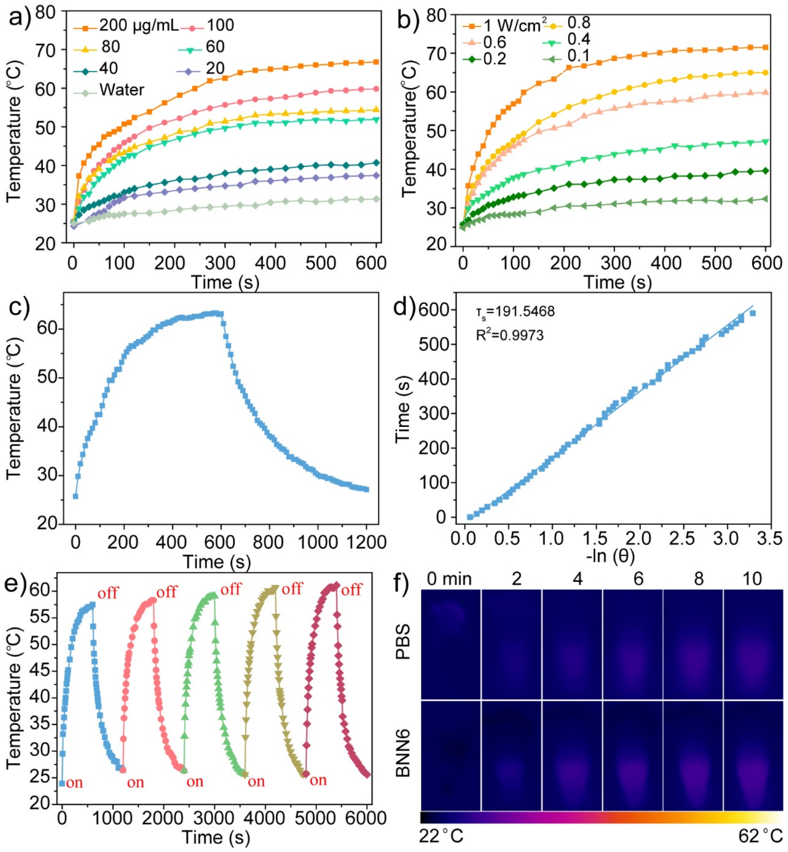

Figure S9. a) Concentration-dependent photothermal curves of CPNPs under 1064 nm laser irradiation. b) Photothermal heating curves of CPNPs dispersions (100 μg/mL) irradiated using a 1064 nm laser at varied power densities (0.1,0.2,0.6,0.8,1.0 W/cm2). c) The photothermal effect of CPNPs aqueous solution (100 μg/mL) excited with 1064 nm laser. d) Linear relationship curves between time (s) versus − ln θ based on panel e. e) Temperature elevation of CPNPs dispersion under five on/off cycles. f) Photothermal IR images of PBS and BNN6 under 1064 nm laser irradiation for 10 min.
